# Akt-elicited phosphorylation of Acapin steers cell migration

**DOI:** 10.1093/jmcb/mjaf010

**Published:** 2025-03-13

**Authors:** Chunyue Wang, Jiajia Zhou, Tao Li, Shihao Du, Tetsuro Urushidani, Dongmei Wang, Yong Chen, McKay Mullen, Xinwang Cao, Shi-Yuan Cheng, Xia Ding, Fengrui Yang, Xuebiao Yao

**Affiliations:** MOE Key Laboratory for Cellular Dynamics, Hefei National Research Center for Physical Sciences at the Microscale, University of Science and Technology of China, Hefei 230027, China; Fuwai Hospital, National Center for Cardiovascular Diseases, State Key Laboratory of Cardiovascular Disease, Chinese Academy of Medical Sciences and Peking Union Medical College, Beijing 100037, China; MOE Key Laboratory for Cellular Dynamics, Hefei National Research Center for Physical Sciences at the Microscale, University of Science and Technology of China, Hefei 230027, China; MOE Key Laboratory for Cellular Dynamics, Hefei National Research Center for Physical Sciences at the Microscale, University of Science and Technology of China, Hefei 230027, China; Department of Gastroenterology, Beijing University of Chinese Medicine, Beijing 100029, China; MOE Key Laboratory for Cellular Dynamics, Hefei National Research Center for Physical Sciences at the Microscale, University of Science and Technology of China, Hefei 230027, China; Department of Gastroenterology, Beijing University of Chinese Medicine, Beijing 100029, China; MOE Key Laboratory for Cellular Dynamics, Hefei National Research Center for Physical Sciences at the Microscale, University of Science and Technology of China, Hefei 230027, China; MOE Key Laboratory for Cellular Dynamics, Hefei National Research Center for Physical Sciences at the Microscale, University of Science and Technology of China, Hefei 230027, China; Departments of Hepatobiliary Surgery and Pathology, Xijing Hospital, Xi'an 710032, China; MOE Key Laboratory for Cellular Dynamics, Hefei National Research Center for Physical Sciences at the Microscale, University of Science and Technology of China, Hefei 230027, China; Atlanta Clinical & Translational Science Institute, Atlanta, GA 30310, USA; MOE Key Laboratory for Cellular Dynamics, Hefei National Research Center for Physical Sciences at the Microscale, University of Science and Technology of China, Hefei 230027, China; The Ken & Ruth Davee Department of Neurology, Northwestern University Feinberg School of Medicine, Chicago, IL 60611, USA; Department of Gastroenterology, Beijing University of Chinese Medicine, Beijing 100029, China; MOE Key Laboratory for Cellular Dynamics, Hefei National Research Center for Physical Sciences at the Microscale, University of Science and Technology of China, Hefei 230027, China; Atlanta Clinical & Translational Science Institute, Atlanta, GA 30310, USA; MOE Key Laboratory for Cellular Dynamics, Hefei National Research Center for Physical Sciences at the Microscale, University of Science and Technology of China, Hefei 230027, China

**Keywords:** cell migration, EGF, Akt, ACAP4, Acapin, ARF6 activity

## Abstract

Cell migration requires the generation of branched actin networks and recruitment of vesicular membrane that power the protrusion of the plasma membrane in lamellipodia. However, the molecular mechanisms underlying dynamic recruitment of vesicular membrane during cell migration remain elusive. Here, we report a critical mechanism underlying epidermal growth factor (EGF)-elicited Akt signaling-steered cell migration. Using functional proteomics screen, we identified a novel ADP-ribosylation factor 6 (ARF6)–ACAP4 signaling regulator, Acapin, which inhibits the GTPase-activating protein (GAP) activity of ACAP4 to activate ARF6 GTPase *in vitro*. In cells, EGF stimulation elicits Akt signaling, which recruits Acapin to the lamellipodium membrane via phosphorylation of Acapin at its Ser247 residue and enhances the binding of Acapin to ACAP4 to elevate the ARF6-GTP level. Therefore, Acapin is required for efficiently stimulating cell migration by EGF–Akt signaling. Together, our results demonstrate the role of Acapin in relaying the Akt signaling cascade during cell migration processes.

## Introduction

Cell migration requires the generation of branched actin networks and recruitment of vesicular membrane that power the protrusion of the plasma membrane in lamellipodia (Fang et al., 2006; Song et al., 2020). ADP-ribosylation factor 6 (ARF6) is an important member of the ARF family of the Ras superfamily of small GTPases. Like other small GTPase, the cycling of ARF6 between the active GTP-bound and inactive GDP-bound states, which is orchestrated by the GTPase-activating proteins (GAPs) and the guanidine nucleotide (GTP–GDP) exchanging factors (GEFs), regulates the intracellular membrane trafficking of ARF6 (Donaldson and Jackson, 2011; Wei et al., 2023). ARF6 GTPase activity is dynamically regulated by its GEFs and GAPs in response to extracellular cues via membrane–cytoskeletal remodeling (Matsukawa et al., 2003; Randazzo et al., 2007; Ding et al., 2010). Previous studies indicated that epidermal growth factor (EGF) receptor (EGFR) induces GEFs to activate ARF6 in invasive cancers, which promotes cancer cell migration and invasion (Morishige et al., 2008).

The spatiotemporal dynamics of ARF6 activity is orchestrated by its GAPs, which assemble signaling scaffold at the plasma membrane (Spang et al., 2010; Mizuno-Yamasaki et al., 2012). ACAP4 (also known as DDEFL1, UPLC1, and ASAP3) is a selective ARF6-specific GAP (Fang et al., 2006; Ismail et al., 2010). ACAP4 regulates membrane–cytoskeletal dynamics in response to a variety of stimuli such as EGF (Fang et al., 2006; Song et al., 2018) and histamine (Ding et al., 2010; Yuan et al., 2017) and is implicated in cancer cell migration and invasion (Okabe et al., 2004; Ha et al., 2008; Song et al., 2018; Mullen et al., 2021). However, how ACAP4 orchestrates the spatiotemporal dynamics of ARF6 GTPase in response to extracellular cues remains elusive.

Akt is an important regulator in control of cell fate and cellular plasticity that are associated with tumor cell migration and invasion (Qiao et al., 2008; Feng et al., 2014). In response to EGF, activated PI3K increases the production of PIP3, which recruits Akt to the plasma membrane, where Akt is activated via phosphorylation (Hemmings and Restuccia, 2012; Feng et al., 2014). Akt is locked at an active conformation and phosphorylates substrates that contain a minimal consensus sequence of RxRxxS/T to promote cellular dynamics and survival (Scheid and Woodgett, 2001). The array of Akt kinase substrates provides an essential survival signal to invasive and metastatic tumor cells (Manning and Cantley, 2007). However, the mechanisms of action underlying Akt-elicited tumor progression have not been fully characterized.

Our previous study showed that the hyperphosphorylation of the ACAP4-binding protein ezrin was closely correlated to an invasive phenotype of clinical hepatocellular carcinomas (HCC) and poor survival in mice with HCC tumor xenografts (Chen et al., 2011). Given the aberrant expression and modification of ACAP4 and ezrin in cancer pathogenesis, we further investigated the mechanism underlying ACAP4-mediated cancer cell invasion and migration. We conducted functional proteomics screen and identified a novel ACAP4-binding protein, which was named Acapin (ACAP4 inhibitor). Acapin binds to the GAP domain of ACAP4, which protects ARF6-GTP from hydrolysis. Interestingly, EGF-activated Akt induces the phosphorylation of Acapin at its Ser247 residue (Acapin^S247^), which promotes ARF6 GTPase activity for powering cancer cell migration.

## Results

### Identification of Acapin, a novel interactor of ACAP4

**Figure 1:**
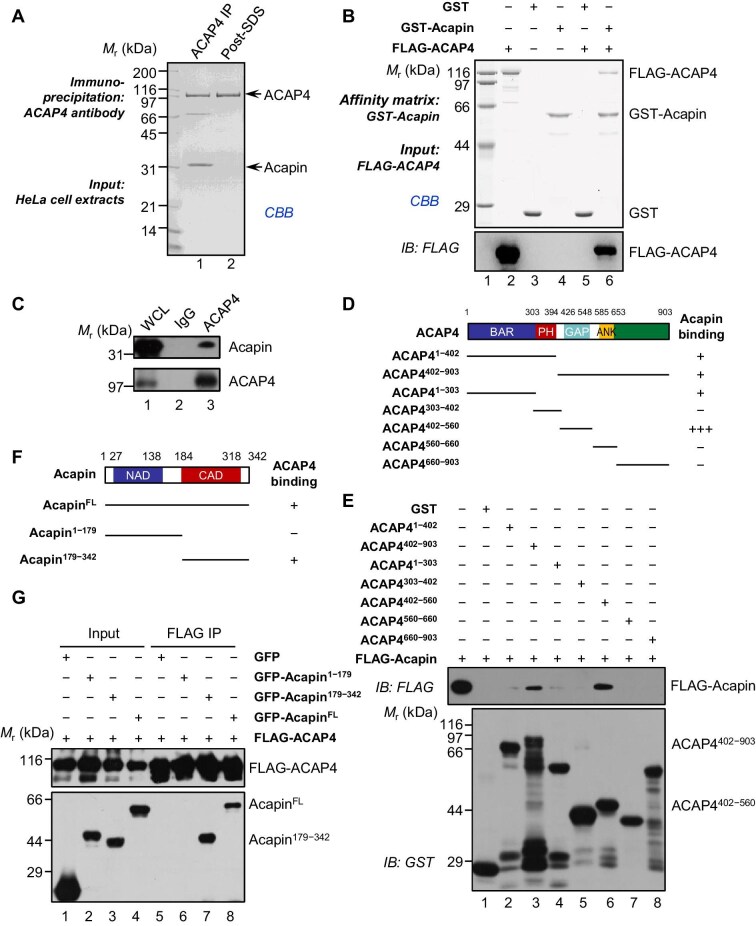
Identification of Acapin as a novel ACAP4-interacting protein. (**A**) IP assay of HeLa cell extracts using an anti-ACAP4 antibody. The immunoprecipitates were eluted using a buffer with or without 0.1% SDS and subjected to SDS–PAGE and CBB staining. (**B**) ACAP4 interacts with Acapin *in vitro*. Recombinant GST-Acapin proteins purified on glutathione beads were used as the affinity matrix for absorbing FLAG-ACAP4. GST-bound glutathione beads were used as a negative control. After washing, proteins bound to the beads were resolved by SDS–PAGE and analyzed by CBB staining and IB using an anti-FLAG antibody. (**C**) IP assay of the endogenous Acapin/ACAP4 protein complex in HeLa cells using an anti-ACAP4 polyclonal antibody or preimmune IgG. (**D**) Schematic drawing of ACAP4 deletion mutants. +++, positive; +, weak; −, negative. (**E**) Acapin binds to the GAP domain of ACAP4. ACAP4 proteins containing different domains as indicated were purified on glutathione beads and used as the affinity matrix for absorbing full-length FLAG-Acapin protein. GST bound glutathione beads were used as a negative control. Proteins bound to the beads were analyzed by IB using an anti-FLAG antibody. (**F**) Schematic drawing of Acapin deletion mutants. +, positive; −, negative. (**G**) The C-terminal arrestin domain of Acapin is responsible for ACAP4 binding. FLAG-ACAP4-bound agarose beads were used as the affinity matrix for absorbing different GFP-tagged Acapin proteins. The immunoprecipitates were analyzed by IB using anti-GFP and anti-FLAG antibodies. Blots shown are representative of two independent experiments with similar results.

To identify potential novel interactors of ACAP4 that is highly expressed in human cancers (Okabe et al., 2004; Fang et al., 2006; Chen et al., 2011), we generated an affinity matrix covalently coupled to an anti-ACAP4 antibody and carried out a proteomic search for proteins that bind to ACAP4 in HeLa cells (Fang et al., 2006). To distinguish the putative ACAP4-specific binding from nonspecific protein binding, proteins were eluted from anti-ACAP4 affinity columns in a buffer with or without 0.1% sodium dodecyl sulphate (SDS), followed by SDS–polyacrylamide gel electrophoresis (SDS–PAGE) and Coomassie Brilliant Blue (CBB) staining. As shown in Figure 1A, three major protein bands including ACAP4, a 70-kDa protein, and a 38-kDa protein were found reproducibly enriched on the ACAP4 matrix before 0.1% SDS wash. The 70-kDa band was identified as ezrin by mass spectrometry and the ACAP4–ezrin interactions were characterized previously (Fang et al., 2006; Ding et al., 2010).

Then, these major protein bands were removed from the acrylamide gel and digested in-gel by trypsin. The resulting peptide fragments of the 38-kDa protein were extracted and analyzed by mass spectrometry, and a previously uncharacterized open reading frame of A6NEK1 (gene ID: 325511327) was identified. A6NEK1 encodes a 342-amino acid protein with unknown function. *In silico* analyses predicted that the protein contains two putative arrestin-like domains and three coiled-coil regions (Supplementary Figure S1A). Because A6NEK1 exhibits unique biochemical function in inhibiting the GAP activity of ACAP4, we named this protein as Acapin.

To establish the biochemical interaction of ACAP4 with Acapin, we used anti-FLAG affinity beads to isolate proteins binding to FLAG-Acapin from lysates of HEK293T cells co-expressing FLAG-Acapin and HA-ACAP4. As shown in Supplementary Figure S1B, immunoblotting (IB) analyses using anti-FLAG and anti-HA antibodies identified an association of ACAP4 with Acapin. To examine whether ACAP4 directly interacts with Acapin, we carried out the pull-down assay using GST-Acapin as an affinity matrix to absorb FLAG-ACAP4 protein purified from HEK293T cells. As shown in Figure 1B, FLAG-ACAP4 was absorbed by GST-Acapin, but not by GST alone, indicating that Acapin is a novel ACAP4-binding protein. The interaction between ACAP4 and Acapin was further demonstrated by yeast two-hybrid assays (Supplementary Figure S1C). Moreover, co-immunoprecipitation (co-IP) studies revealed that endogenous ACAP4 interacted with endogenous Acapin in HeLa cells (Figure 1C). ACAP4 is a signaling scaffold molecule containing several structure modules for orchestration of protein–protein interactions underlying signaling cascade during cell polarity establishment and cell migration (Fang et al., 2006; Ismail et al., 2010; Zhao et al., 2013; Song et al., 2018). To define the binding interface between ACAP4 and Acapin, we designed a series of deletion mutants of ACAP4 according to its structural features (Figure 1D) and expressed these truncation mutants as GST fusion proteins. We then carried out the pull-down assay using various GST-ACAP4 proteins as the affinity matrix to absorb FLAG-Acapin expressed by HEK293T cells. As shown in Figure 1E, Acapin was firmly retained by the GAP domain in ACAP4 proteins (amino acids 402−560, lane 6), while the BAR domain (amino acids 1−303, lane 4) and the PH domain (amino acids 303–402, lane 5) exhibited a much weaker interaction with Acapin.

There are several ARF6 GAPs that regulate ARF6 GTPase activity (Gillingham and Munro, 2007). We next examined whether other known ARF6 GAPs bind to Acapin. As shown in Supplementary Figure S1D, ACAP1 (Ma et al., 2007), ACAP2 (Jackson et al., 2000), and ARAP3 (Krugmann et al., 2002) did not exhibit any binding activity toward Acapin, suggesting a relatively selective interaction of Acapin with ACAP4 due to divergent sequences of the ARF6 GAP domains. Conversely, to pinpoint the region of Acapin involved in its binding to ACAP4, we generated a series of Acapin deletion mutants tagged with GFP (Figure 1F). Aliquots of HEK293T cells were transiently transfected to express FLAG-ACAP4 with various GFP-Acapin constructs. FLAG IP followed by IB analyses using an anti-GFP antibody demonstrated that the C-terminus of Acapin protein mediated the Acapin–ACAP4 interaction (Figure 1G, lane 7). Thus, these data indicate that Acapin interacts with the GAP domain of ACAP4 through its C-terminal arrestin domain.

### Acapin is an endogenous ACAP4 inhibitor and promotes ARF6 GTPase activity

**Figure 2:**
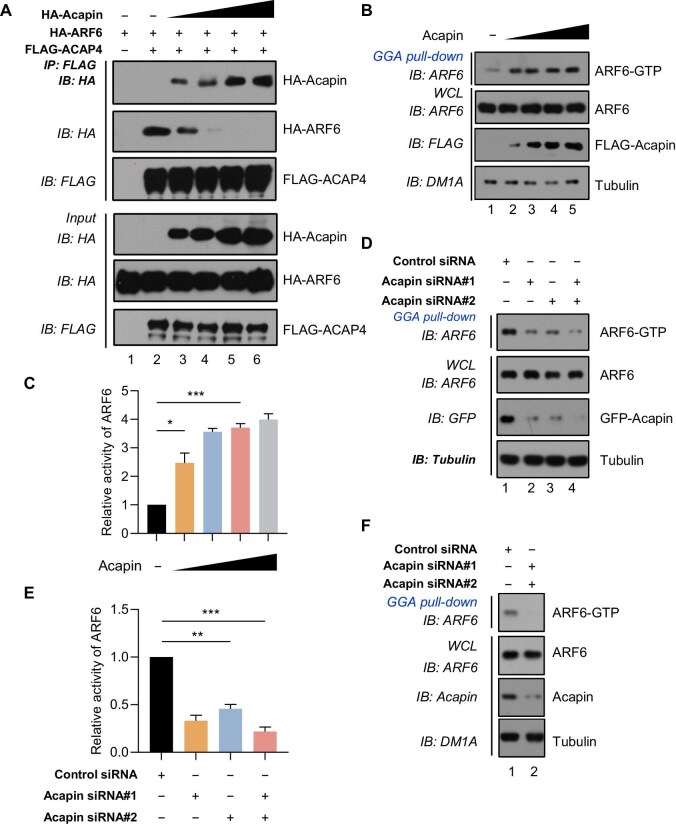
Acapin promotes ARF6 GTPase activity via inhibiting the ACAP4–ARF6 interaction. (**A**) Acapin competes with ARF6 for binding to ACAP4. HEK293T cells were co-transfected with HA-ARF6 and FLAG-ACAP4 in the presence of increasing amounts of HA-Acapin (wedges). The cells were lysed in IP buffer containing 100 μM GTPγS and subjected to FLAG IP and IB with the indicated antibodies. (**B** and **C**) Dose-dependent effect of Acapin on ARF6 activity. HeLa cells overexpressing FLAG-Acapin were subjected to GGA pull-down assay and IB with the indicated antibodies, followed by statistical analysis of relative ARF6 activity. Data are presented as mean ± SEM, *n =* 3, *P < 0.05, ***P < 0.001. (**D** and **E**) Acapin is required for ARF6 activity. HeLa cells transfected with either or both siRNAs targeting Acapin were subjected to GGA pull-down assay and IB, followed by statistical analysis of relative ARF6 activity. Data are presented as mean ± SEM, *n =* 3, **P < 0.01, ***P < 0.001. (**F**) Acapin depletion inhibits ARF6 activity. HeLa cells were transfected with an Acapin-targeting siRNA for 24 h (initial transfection) and received a second round of transfection with another Acapin-specific siRNA, followed by GGA pull-down assay to assess the level of active ARF6-GTP.

Previous studies reported that ARF6 interacts with the GAP domain of ACAP4 (Fang et al., 2006; Ismail et al., 2010). The interaction of Acapin with the GAP domain of ACAP4 prompted us to examine whether Acapin competes with ARF6 for binding to ACAP4. To this end, HA-Acapin was transiently transfected into HEK293T cells together with HA-ARF6 and FLAG-ACAP4. To prevent the inactivation of wild-type ARF6 by ACAP4 and subsequent dissociation of the complex, cell lysates were pretreated with 100 μM GTPγS, a nondegradable GTP analog. As shown in Figure 2A, ARF6 bound to ACAP4 in the absence of Acapin, but increasing amounts of Acapin protein liberated ARF6 from ACAP4 binding, suggesting that Acapin binds to ACAP4 in a higher affinity than ARF6 and Acapin disrupts the ACAP4–ARF6 interaction by replacing ARF6 in the GAP domain of ACAP4. Similarly, when HEK293T cells were transfected with constitutively active ARF6^Q67L^, HA-Acapin, and FLAG-ACAP4, overexpression of HA-Acapin diminished the binding ability of FLAG-ACAP4 to HA-ARF6^Q67L^ (Supplementary Figure S2A). To assess whether Acapin releases ARF6 in GTP-bound form from ACAP4 binding, we carried out GGA pull-down assay using a GST fusion protein containing the VHS and ARF binding domains of GGA3 (GST-GGA) that only recognizes GTP-bound active ARF6 (ARF6-GTP). As shown in Figure 2B, expression of increasing amounts of Acapin protein markedly increased ARF6-GTP contents while not altering ARF6 protein expression, suggesting that Acapin protects ARF6-GTP from ACAP4-elicited hydrolysis. Furthermore, the ARF6 GTPase activity by quantifying the relative ARF6-GTP level over ARF6 protein level was a function of Acapin expression level in cells (Figure 2C).

Next, we carried out siRNA-mediated inhibition of Acapin. As shown in Supplementary Figure S2B, transient transfection of two independent siRNAs into cells resulted in an appreciable decrease in Acapin protein expression. HeLa cells stably expressing GFP-Acapin were transfected with two independent Acapin siRNAs, separately or in combination, followed by GGA pull-down assays. As shown in Figure 2D, siRNA-mediated knockdown of Acapin resulted in a marked reduction in ARF6-GTP content. Again, the relative activity of ARF6 was a function of Acapin protein level in cells (Figure 2E). Similarly, siRNA-mediated inhibition of endogenous Acapin in HeLa cells resulted in a considerable reduction in ARF-GTP content but did not alter the expression level of ARF6 (Figure 2F) and thus significantly reduced the ARF6 GTPase activity (Supplementary Figure S2C). Collectively, these results indicate that Acapin promotes ARF6 GTPase activity by interfering the ACAP4–ARF6 interaction.

### Phosphorylation of Acapin by Akt enhances the Acapin–ACAP4 interaction

**Figure 3:**
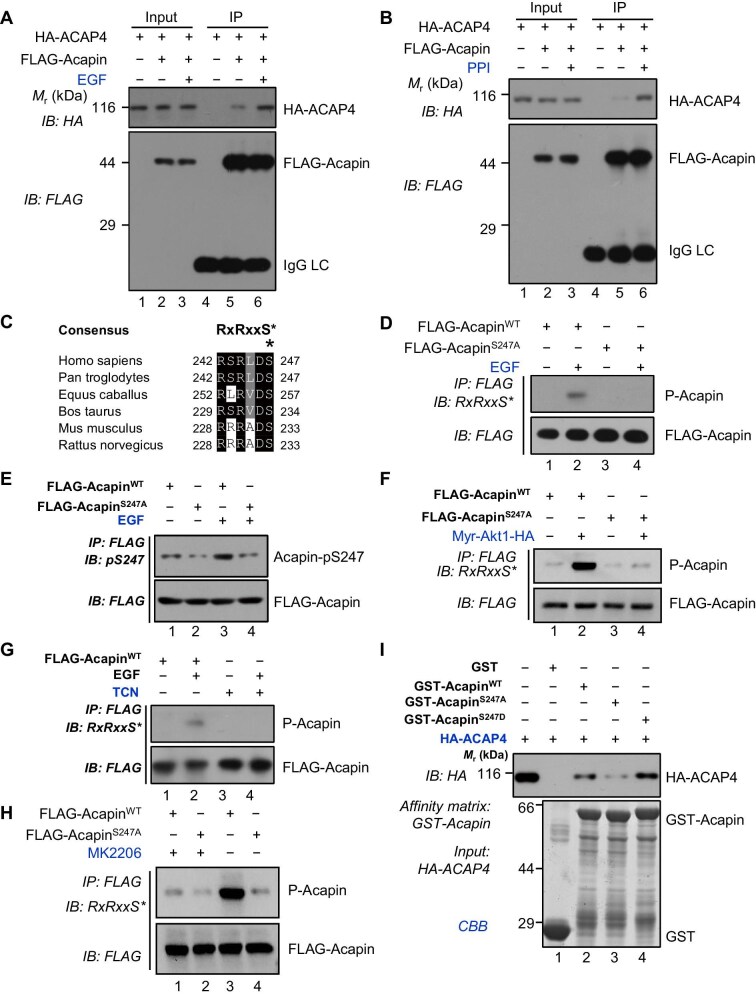
EGF induces Acapin phosphorylation and its interaction with ACAP4. (**A**) EGF stimulation enhances the association of Acapin with ACAP4. HeLa cells were transfected with FLAG-Acapin and HA-ACAP4. After 24 h, the cells underwent serum starvation for 6 h, followed by stimulation with EGF (100 ng/ml) for 5 min. The cell extracts were subjected to FLAG IP and IB with anti-HA and anti-FLAG antibodies. (**B**) Phosphatase inhibitor PPI treatment increases the interaction between Acapin and ACAP4. HeLa cells co-expressing FLAG-Acapin and HA-ACAP4 were treated with PPI for 2 h, followed by co-IP using anti-FLAG M2 beads and IB analysis. (**C**) Alignment of the amino acid sequences of the canonical Akt kinase phosphorylation motif in Acapin across *Homo sapiens, Pan troglodytes, Equus caballus, Bos taurus, Mus musculus*, and *Rattus norvegicus*. Dark and light shading indicate identical and conserved residues, respectively. (**D** and **E**) FLAG-Acapin^WT^ and the nonphosphorylatable mutant FLAG-Acapin^S247A^ were expressed in HeLa cells. Serum-starved cells were stimulated by EGF (100 ng/ml) and lysed for FLAG IP, followed by IB analyses using an anti-Akt-pSub antibody (indicating phosphorylated Acapin, annotated as P-Acapin) and an anti-FLAG antibody, respectively (**D**) or using an anti-Acapin-pS247 antibody and an anti-FLAG antibody, respectively (**E**). (**F**) *In vitro* kinase assay. FLAG-Acapin^WT^ and FLAG-Acapin^S247A^ were co-transfected with a constitutively active form of Akt (Myr-Akt1) into HeLa cells. Akt-elicited phosphorylation was determined by IB using an anti-Akt-pSub antibody. (**G**) Serum-starved HeLa cells expressing FLAG-Acapin were treated with TCN for 2 h followed by EGF (100 ng/ml) stimulation for 5 min. The phosphorylation of Acapin was determined by FLAG IP and IB using an anti-Akt-pSub antibody. (**H**) The phosphorylation of Acapin was determined by FLAG IP and IB using an anti-Akt-pSub antibody in HeLa cells expressing FLAG-Acapin^WT^ or FLAG-Acapin^S247A^ with or without MK2206. (**I**) Phosphorylation at Acapin^S247^ is required for Acapin to interact with ACAP4. GST-Acapin^WT^, GST-Acapin^S247A^, and GST-Acapin^S247D^ on glutathione beads were incubated with HA-ACAP4 from HEK293T cells. After washing, proteins bound to the beads were analyzed by CBB staining and IB using an anti-HA antibody.

Our previous work revealed that EGF elicits cancer metastasis and ACAP4 orchestrates EGF-elicited cellular dynamics (Fang et al., 2006; Zhao et al., 2013; Song et al., 2018). To examine whether EGF stimulation modulates the ACAP4–Acapin interaction in cells, HeLa cells were transiently co-transfected with FLAG-Acapin and HA-ACAP4, serum-starved for 6 h, and stimulated with EGF (100 ng/ml) for 5 min. FLAG IP and IB analyses indicated that the Acapin–ACAP4 association in HeLa cells was enhanced by EGF stimulation (Figure 3A). This was further confirmed in HeLa cells co-transfected with FLAG-ACAP4 and HA-Acapin and stimulated with EGF (Supplementary Figure S3A).

Mounting evidence has established the critical role of EGFR–Akt signaling in promoting cancer cell invasion and migration (Leemans et al., 2011). To examine whether EGF-elicited phosphorylation modulates the Acapin–ACAP4 interaction, HeLa cells were transiently transfected with HA-ACAP4 and FLAG-Acapin followed by treatment with phosphatase inhibitor cocktail 1 (PPI1) and subjected to IP–IB analyses. A greater retention of HA-ACAP4 on FLAG-Acapin beads was observed in PPI1-treated cells when compared to the nontreated cells (Figure 3B, lane 6 vs. lane 5), suggesting that protein phosphorylation promotes the Acapin–ACAP4 interaction. *In silico* analyses using a GPS software (Xue et al., 2005) identified the amino acid sequence surrounding Ser247, RSRLDS (amino acids 242–247), in Acapin polypeptide as a putative Akt substrate, since this fragment contains a characteristic motif RxRxxS for Akt phosphorylation (Zhang et al., 2002). Interestingly, sequence alignment analyses indicated that this motif is conserved among several species (Figure 3C).

To experimentally determine whether Acapin^S247^ is a *bona fide* substrate for Akt phosphorylation, HeLa cells were transfected with wild-type FLAG-Acapin (FLAG-Acapin^WT^) or a nonphosphorylatable FLAG-Acapin^S247A^ mutant and subjected to IP–IB analyses. The phosphorylation of Acapin^S247^ was scored by a well-characterized anti-phospho-Akt substrate antibody (anti-Akt-pSub, annotated as P-Acapin), which reacts with the RxRxx*p*S motif (Liu et al., 2015). As shown in Figure 3D, EGF-induced phosphorylation of Acapin was readily detectable in FLAG-Acapin^WT^ (lane 2) but not found in FLAG-Acapin^S247A^ (lane 4), suggesting that EGF induces Acapin phosphorylation at Ser247. We also generated an anti-Acapin-pS247 antibody that specifically recognizes a peptide sequence of Acapin containing a phosphorylated Ser247 residue (Supplementary Figure S3B) to determine the phosphorylated Acapin. As shown in Figure 3E, EGF stimulation efficiently induced Acapin phosphorylation at Ser247. Furthermore, HeLa cells were transiently transfected with FLAG-Acapin^WT^ or FLAG-Acapin^S247A^ together with a constitutively active Akt (Myr-Akt1-HA). We found that FLAG-Acapin^WT^ but not the FLAG-Acapin^S247A^ mutant was phosphorylated by Myr-Akt1 (Figure 3F, lane 2 vs. lane 4). Additionally, we expressed FLAG-Acapin^WT^ or FLAG-Acapin^S247A^ in HeLa cells and examined their phosphorylation patterns under asynchronous conditions using phos-tag-PAGE analyses. We observed a band shift in FLAG-Acapin^WT^ but not in FLAG-Acapin^S247A^, suggesting that phosphorylation of Ser247 in recombinant Acapin protein determines the migration change (Supplementary Figure S3C). To establish Akt as a upstream kinase for the phosphorylation of Acapin^S247^, we treated cells with two chemically distinct Akt inhibitors triciribine (TCN) (Figure 3G) and MK2206 (Figure 3H). Chemical inhibition of Akt kinase activity by MK2206 significantly reduced the phosphorylation of Acapin in cells (Figure 3H, lane 1 vs. lane 3). Collectively, these data show that Acapin^S247^ is a cognate substrate of Akt kinase in response to EGF stimulation.

Next, we examined whether Akt-induced phosphorylation of Acapin^S247^ modulates Acapin–ACAP4 association. As shown in Figure 3I, a phospho-mimicking mutant Acapin^S247D^ robustly absorbed HA-ACAP4 (lane 4), whereas the nonphosphorylatable Acapin^S247A^ mutant had a markedly weaker binding to ACAP4 (lane 3). Similarly, recombinant Acapin^S247D^ exhibited a stronger binding to the GAP domain of ACAP4 (ACAP4^402–560^) whereas Acapin^S247A^ displayed a weaker association with ACAP4^402–560^ (Supplementary Figure S3D). These data suggest that Akt-induced phosphorylation of Acapin^S247^ promotes the association of Acapin with the GAP domain of ACAP4.

Previous data indicated that EGF also induces Tyr34 and Tyr733 phosphorylation of ACAP4 (Yu et al., 2011; Zhao et al., 2013). However, EGF-induced phosphorylation of Tyr34 or Tyr733 did not influence the ACAP4–Acapin interaction (Supplementary Figure S4A and B). Additionally, we conducted a molecular modeling of ARF6–ACAP4 binding interface and identified a positively charged region in the GAP domain of ACAP4 as previously reported (Huang et al., 2019; Song et al., 2021). The region is composed of Arg469, Arg474, and Arg477 (Supplementary Figure S4C), which has been reported to participate in the ARF6–ACAP4 binding (Ismail et al., 2010; Zhao et al., 2013). Thus, it is plausible that the electrostatic interaction elicited by the phosphorylation of Acapin^S247^ enhances the negatively charged Acapin binding to the positively charged GAP domain of ACAP4.

### Ser247 phosphorylation of Acapin by Akt kinase promotes ARF6 activity

**Figure 4:**
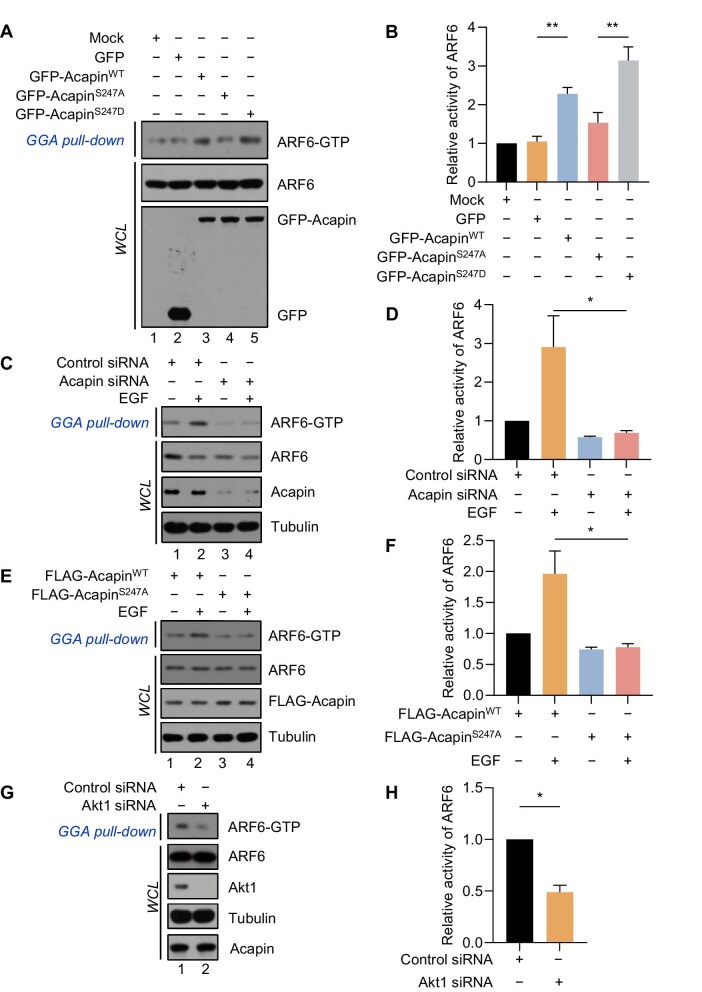
Phosphorylation of Acapin protects ARF6-GTP from ACAP4-elicited hydrolysis. (**A** and **B**) HeLa cells stably expressing GFP, GFP-Acapin^WT^, GFP-Acapin^S247A^, or GFP-Acapin^S247D^ were subjected to GGA pull-down assay and IB with the indicated antibodies, followed by statistical analysis of relative ARF6 activity. Data are presented as mean ± SEM, *n =* 3, **P < 0.01. (**C** and **D**) Acapin is essential for ARF6 activation upon EGF stimulation. HeLa cells were transfected with Acapin siRNAs for 48 h, deprived of serum for 6 h, and treated with EGF (100 ng/ml) for 5 min. The relative ARF6 activity was determined. Data are presented as mean ± SEM, *n =* 3, *P < 0.05. (**E** and **F**) Nonphosphorylatable Acapin^S247A^ mutant fails to reverse ACAP4-mediated ARF6 inactivation. The relative ARF6 activity was determined in EGF-stimulated HeLa cells expressing FLAG-Acapin^WT^ or FLAG-Acapin^S247A^. Data are presented as mean ± SEM, *n =* 3, *P < 0.05. (**G** and **H**) Akt is required for Acapin-mediated ARF6 activation. HeLa cells stably expressing GFP-Acapin were treated with Akt1 siRNAs. The relative ARF6 activity in Akt-depleted or control cells was determined. Data are presented as mean ± SEM, *n =* 3, *P < 0.05.

To examine whether the phosphorylation of Acapin^S247^ modulates the ARF6-GTP level, HeLa cells separately expressing GFP, GFP-Acapin^WT^, GFP-Acapin^S247A^, or GFP-Acapin^S247D^ were treated with EGF for 5 min and subjected to GGA pull-down assay. As shown in Figure 4A and B, the ARF6-GTP level and ARF6 activity were significantly higher in cells expressing GFP-Acapin^WT^ and GFP-Acapin^S247D^ mutants than in cells expressing GFP-Acapin^S247A^, suggesting that the perturbed Acapin–ACAP4 interaction by S247A mutation in Acapin promotes ACAP4-mediated hydrolysis of ARF6-GTP. Consistently, siRNA-mediated depletion of Acapin that perturbed the ARF6–ACAP4 interaction also diminished the ARF6-GTP level (Figure 4C; lane 4) and ARF6 GTPase activity (Figure 4D) upon EGF stimulation. Additionally, the overexpression of Acapin^WT^, but not the nonphosphorylatable Acapin^S247A^ mutant, promoted the ARF6-GTP level and ARF6 GTPase activity upon EGF stimulation (Figure 4E and F).

To ascertain that ARF6 GTPase activity is a function of Akt–Acapin signaling, HeLa cells were transiently transfected with siRNAs for Akt and subjected to GGA pull-down assays. As expected, knockdown of Akt by siRNAs attenuated the phosphorylation of Acapin and subsequent ARF6 GTPase activity (Figure 4G and H). These results indicate that the phosphorylation of Acapin^S247^ increases ARF6 GTPase activity by sequestering ACAP4, which protects ARF6-GTP from ACAP4-mediated hydrolysis.

### Acapin promotes the membrane–cytoskeleton remodeling in response to EGF

**Figure 5:**
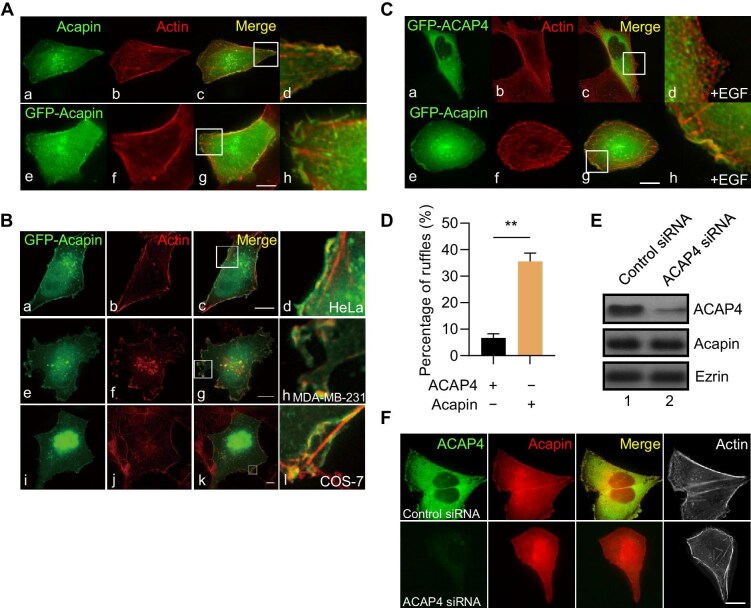
Acapin is critical for EGF-induced cell migration and invasion. (**A**) Plasma membrane localization of Acapin in cells. (a–d) HeLa cells were fixed, permeabilized, and stained for endogenous Acapin (green) and actin (red). (e–h) GFP-Acapin was transfected into HeLa cells. After 24 h, the cells were fixed, permeabilized for visualization of GFP-Acapin (green), and counterstained with phalloidin (Actin, red). Scale bar, 10 μm. Panels d and h are magnified portion (indicated by a box) of merge panels c and g, respectively. (**B**) HeLa, MDA-MB-231, and COS-7 cells were transfected with GFP-Acapin for 24 h and then fixed, permeabilized for visualization of GFP-Acapin (green), and counterstained with phalloidin (Actin, red). Scale bar, 10 μm. Panels d, h, and l are magnified portion (indicated by a box) of merge panels c, g, and k, respectively. (**C** and **D**) HeLa cells were transfected with GFP-ACAP4 (a–d) and GFP-Acapin (e–h), respectively. After 24 h, the cells were deprived of serum for 6 h, followed by EGF stimulation for 5 min, and then fixed and stained with phalloidin to detect actin (red). Scale bar, 10 μm. Protrusions of the cells were quantified. Data are presented as mean ± SEM, **P < 0.01. (**E** and **F**) Depletion of ACAP4 by siRNAs abolishes the plasma membrane localization of Acapin without alternating actin filament localization in HeLa cells.

ARF6 functions in membrane–cytoskeletal dynamics at the cell periphery (Schweitzer et al., 2011). Given that Akt-mediated phosphorylation of Acapin enhances ARF6 activity, we next investigated its effects on cellular dynamics and motility. To this end, we carried out dual-color immunofluorescence microscopic analyses to examine the distribution of Acapin and ACAP4 as well as filamentous actin in cells. As shown in Figure 5A, both endogenous Acapin and exogenously expressed GFP-Acapin were co-localized with a subset of filamentous actin marked by phalloidin in HeLa cells. Similar distribution patters were observed in MDA-MB-231 and COS-7 cells (Figure 5B). The majority of cells exhibited a cell periphery membrane distribution of Acapin. In the co-transfected cells, Acapin and ACAP4 were also co-localized at the plasma membrane (Supplementary Figure S5A). Furthermore, Acapin was co-localized with both Arp3 (a component of the actin nucleation machinery) and ezrin (a plasma membrane–microfilament linker protein) (Supplementary Figure S5B).

Next, we examined the interplay between Acapin and ACAP4 at the cell plasma membrane. As shown in Figure 5C and D, membrane ruffles in HeLa cells expressing GFP-Acapin were readily apparent upon EGF stimulation, indicating that Acapin promotes membrane ruffle formation in response to EGF. Furthermore, we carried out siRNA-mediated knockdown of ACAP4 and examined Acapin distribution in cells. The siRNA-mediated depletion of ACAP4 abolished the plasma membrane localization of Acapin without affecting that of actin filament (Figure 5E and F), suggesting that ACAP4 is responsible for Acapin localization to the membrane ruffles.

### Ser247 phosphorylation of Acapin by Akt promotes directional cell motility

**Figure 6:**
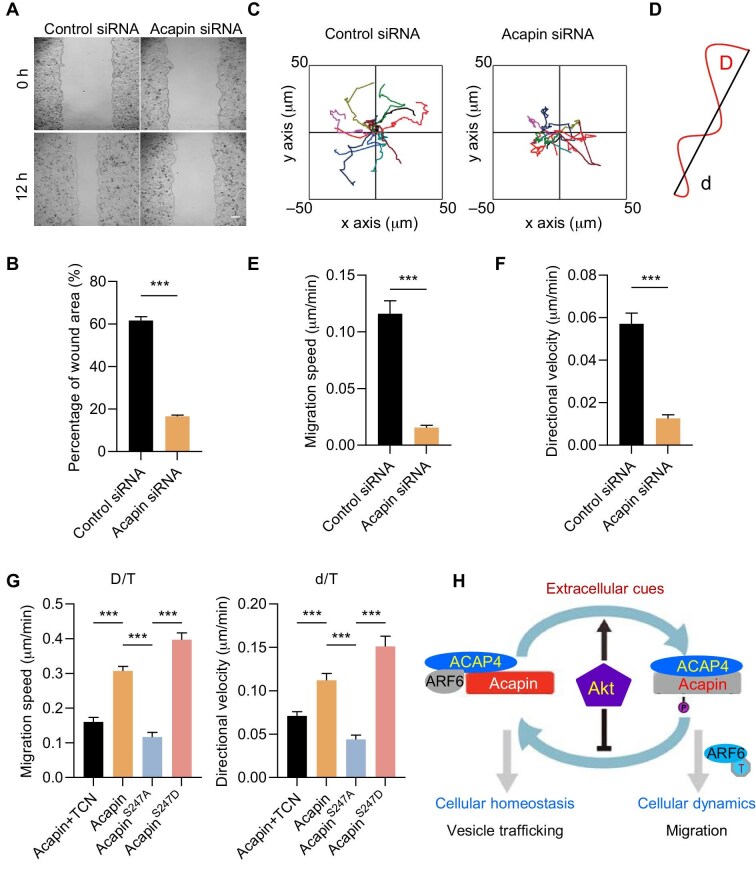
Akt-induced phosphorylation of Acapin^S247^ is required for efficient cell motility *in vitro*. (**A** and **B**) Acapin depletion inhibits cell migration for wound healing. MDA-MB-231 cells transfected with Acapin siRNAs were subjected to the wound healing assay, followed by quantification of the relative wound area toward the opposite sides. Scale bar, 100 μm. Data are presented as mean ± SEM from three independent experiments. ***P < 0.001 by Student's *t*-test. (**C**–**F**) MDA-MB-231 cells were transfected with Acapin siRNAs for 48 h, serum-starved for 6 h, and then stimulated with EGF (100 ng/ml) for 5 min. (**C**) The tracks of six randomly picked cells for each group. (**D**) Visual demonstration of path length showing the distance between starting and ending points (d) and the actual trajectory (D). (**E** and **F**) Quantification of cell migration speed (D/T) and directional velocity (d/T). Data are presented as mean ± SEM. ***P < 0.001 by Student's *t*-test. (**G**) Cell migration speed and directional velocity of MDA-MB-231 cells expressing GFP-Acapin^WT^ (with or without TCN treatment), GFP-Acapin^S247A^, or GFP-Acapin^S247D^. Data are presented as mean ± SEM from three independent experiments. Ordinary one-way ANOVA followed by Tukey's post hoc test, ***P < 0.001. (**H**) A working model illustrating the mechanism through which Akt-elicited Acapin phosphorylation regulates ACAP4–ARF6 interactions during cell migration.

We explored the role of Acapin in cell motility by performing wound healing assays in MDA-MB-231 cells (Fang et al., 2006; Song et al., 2018). The inhibition of Acapin by siRNAs slowed down cell migration to seal the wound (Figure 6A), which was validated by statistical analyses of three independent experiments (Figure 6B).

Single cell trajectories illustrated that Acapin-depleted cells explored a narrower territory compared with control cells (Figure 6C), suggesting defects in directional migration (Figure 6D). Quantitative analyses of three independent experiments showed that the depletion of Acapin significantly reduced the average migration speed and directional velocity, respectively (Figure 6E and F). Immunoluorescence microscopic examination revealed that depletion of Acapin suppressed cell protrusions upon EGF stimulation (Supplementary Figure S6A), reminiscent of the observation in aluminium fluoride-stimulated cells with elevated ARF6 GTPase activity (Radhakrishna et al., 1996). Therefore, these results indicate that Acapin is essential for the EGF-elicited membrane–cytoskeleton reorganization underlying cell migration.

Since EGF-activated Akt phosphorylates Acapin^S247^, we sought to determine the subcellular distribution of Acapin upon EGF stimulation in the presence or absence of the Akt inhibitor MK2206 or TCN. As shown in Supplementary Figure S6B, membrane ruffles were readily detectable in response to EGF stimulation, but MK2206 inhibition of Akt abolished the location of Acapin to the plasma membrane. Further experiments revealed that membrane ruffles were readily apparent upon EGF stimulation in cells expressing FLAG-Acapin but abolished by TCN pretreatment, and membrane ruffles were greatly enhanced in cells expressing the phospho-mimicking Acapin^S247D^ mutant but hardly detectable in cells expressing the nonphosphorylatable Acapin^S247A^ mutant (Supplementary Figure S6C and D). Quantitative analyses confirm the essential role of Akt-induced phosphorylation of Acapin^S247^ in response to EGF-stimulated membrane remodeling (Supplementary Figure S6D). Moreover, membrane ruffles were similarly detected in cells co-transfected with Acapin^WT^ or Acapin^S247D^ and ACAP4 upon EGF stimulation, while TCN treatment inhibited the formation of ruffles (Supplementary Figure S6E and F). These data indicate that Akt-induced phosphorylation of Acapin promotes Acapin-mediated membrane dynamics and directional migration in response to EGF stimulation.

Several studies have indicated that the Akt1 signaling pathway is aberrantly upregulated in tumors and plays a key role in cancer metastasis (Chen et al., 2011). We then monitored the trajectories of MDA-MB-231 cells seeded on fibronectin-coated Petri dishes by real-time imaging. As shown in Figure 6G, cells expressing GFP-Acapin^WT^ or GFP-Acapin^S247D^ exhibited significantly higher migration speed and directional velocity than the cells expressing GFP-Acapin^S247A^, and TCN treatment significantly reduced the migration speed and directional velocity of the cells expressing GFP-Acapin^WT^. Therefore, we conclude that Akt-induced phosphorylation of Acapin^S247^ guides cell migration in response to EGF stimulation.

We next examined the role of Acapin in EGF-elicited cell invasion using the Boyden chamber assay as previously reported (Chen et al., 2011; Song et al., 2018). Specifically, HeLa cells transiently transfected with Acapin siRNAs or the scramble control were added to the Boyden chamber, stimulated with 100 ng/ml of EGF or phosphate-buffered saline (PBS) as control, and imaged to count the cells passing through the Boyden chamber. As shown in Supplementary Figure S7A, the depletion of Acapin dramatically suppressed the invasion, as fewer Acapin-deficient cells passed through the Boyden chamber. These results indicate that Acapin facilitates EGF-elicited cell invasion.

## Discussion

ARF6 activation operates through a molecular switch mechanism that is spatially and temporally regulated (Pasqualato et al., 2001). While the downregulation of ARF6 activation signaling via ARF6 GAPs plays a crucial role in controlling various ARF6-dependent cellular processes (Jackson et al., 2000; Yu et al., 2011; Song et al., 2018), the regulation of ARF6 GAPs themselves, including potential unidentified regulators, remains poorly understood. In this study, we discovered that Acapin acts as a novel regulator of the ARF6 GAP, ACAP4, thereby facilitating ARF6 activation. Acapin is essential for the efficient promotion of cell migration by EGF, through Akt-mediated phosphorylation at Ser247. This finding suggests that the EGFR–Akt–Acapin axis constitutes a critical regulatory pathway in cell migration and invasion.

We suggest that Acapin is essential for the migratory activity mediated by ACAP4 and plays a crucial role in bridging growth factor signaling with membrane–cytoskeletal dynamics. On one side, the interaction of Acapin with ACAP4 is necessary for sustaining the cycling of ARF6 GTPase during the homeostatic processes of vesicular membrane trafficking (Figure 6H). On the flip side, Acapin, upon Ser247 phosphorylation by Akt, enhances ARF6 GTPase activity and fosters the interaction between the actin-based cytoskeleton and the plasma membrane (Figure 6H). This concept is further supported by our proteomic discovery of the membrane–cytoskeleton linker ezrin within the ARF6/ACAP4 protein complex. Future studies will aim to clarify the mechanisms through which Acapin engages with other membrane–cytoskeletal proteins found in the ACAP4 complex (Fang et al., 2006; Song et al., 2021). In addition, it would be of great interest to examine how Acapin retains its selective interaction with ACAP4 but not other ARF6 GAPs, given the high percentage of conservation in amino acids. A combination of biochemical characterization with real-time analysis of the molecular dynamics involving Acapin–ACAP4–ARF6 will help us to integrate these protein–protein interactions into a comprehensive model. This model will elucidate the signaling pathways of the ARF6/ACAP4/Acapin protein complex in cell migration and invasion.

Our findings indicate that the phosphorylation of Acapin by Akt at the RxRxxS motif is pivotal for its localization at the leading edge. The activation of Akt in response to various external stimuli is a significant upstream event in cell migration (Xue and Hemmings, 2013). In this study, we established a strong link between the activation of Akt, the phosphorylation of Acapin, and Acapin localization at the leading edge. We suggest that Acapin anchors ACAP4 at the leading edge to maintain active ARF6 following EGF stimulation. This interaction may enhance or maintain the ARF6 activity, enabling the activation of downstream effectors critical for cell migration. Moreover, an excess of GTP-bound ARF6 could draw ARF6 GAP away from Acapin, thus averting hyperactivation. Without Acapin at the leading edge, ACAP4 elicits the hydrolysis of ARF6, resulting in a rapid deactivation of ARF6 and the inhibition of cell migration. It is worth noting that the phosphorylation of Acapin^S247A^ probed by anti-Akt-pSub (annotated as P-Acapin) was also inhibited by MK2206 (Figure 3H), suggesting the existence of additional Akt substrates in Acapin.

Early research has highlighted the aberrant upregulation of ARF6 in the context of EGF-induced cell migration and invasion (Fang et al., 2006; Hu et al., 2012), yet the underlying mechanisms remain unclear. Our study unveils that the phosphorylation of Acapin^S247^ introduces a novel regulatory avenue through which the EGF–Akt signaling pathway directs ARF6-mediated cell migration. It would be worth exploring the dynamics of relevant proteins in the Acapin/ACAP4/ARF6 complex during single-cell migration in response to EGF stimulation using spectral imaging analyses (Liu et al., 2020). Given the nature of hierarchical interactions identified in the Acapin–ACAP4–ARF6 signaling axis, future investigation will be greatly facilitated by engineering an auxin-induced degron cell line (Huang et al., 2019).

The observation that inhibiting Ser247 phosphorylation of Acapin suppressed cell migration *in vitro* suggests a potential link with cancer cell invasion and progression. Notably, our earlier investigation into cancer invasion and metastasis proposed that the dynamic phosphorylation of ezrin at Thr567 may serve as an initial priming event, preparing the ezrin molecule for further post-translational modifications as the disease progresses (Chen et al., 2011; Du et al., 2020; Song et al., 2020). Given that ACAP4’s interaction with ezrin undergoes context-dependent conformational changes (Ding et al., 2010; Jiang et al., 2015; Song et al., 2018), it would be intriguing to explore whether and how the phosphorylation of ezrin at Ser567 interacts with Acapin phosphorylation to influence cancer metastasis using xenograft in the future.

In sum, Acapin emerges as a novel regulator of ARF6 GTPase, exerting its influence by inhibiting the GAP activity of ACAP4. Moreover, our study demonstrates that Akt-mediated phosphorylation of Acapin^S247^ is pivotal in orchestrating the Acapin–ACAP4–ARF6 signaling axis, thereby directing cell steering for effective migration and invasion in response to EGF stimulation. These insights underscore the critical role of small GTPases and intracellular signaling in the formation of cortical networks. Moving forward, further investigation into the molecular and cellular mechanisms of action by Acapin will shed light on its involvement in cancer pathogenesis, particularly in relation to tumor progression and the development of invasive characteristics.

## Materials and methods

### Cell culture and transfection

HEK293T, HeLa, COS-7, and MDA-MB-231 cells were obtained from ATCC and cultured in Dulbecco's modified Eagle growth medium (DMEM; Gibco) containing 10% fetal bovine serum (FBS; Hyclone), 100 units/ml penicillin (Gibco), and 100 μg/ml streptomycin (Gibco). All cell lines were cultured at 37°C in 5% CO_2_. Plasmids were transfected into cells using Lipofectamine 3000 (Invitrogen) according to the manufacturer's instructions. For FBS deprivation, cells were washed with PBS once and then incubated in FBS-free DMEM for the indicated time.

### Chemicals and antibodies

Phosphatase inhibitor cocktail 1 (P2850) was purchased from Sigma. TCN, Akt signal pathway inhibitor (Akt Inhibitor V), and EGF were from Calbiochem. MK2206 was purchased from Selleck Chemicals LLC. Mouse monoclonal anti-FLAG M2 antibody and tubulin (DM1A) antibody were obtained from Sigma. Rabbit anti-ARF6 antibody, rabbit anti-ezrin antibody, rabbit anti-HA antibody, mouse anti-myc antibody, rabbit anti-Akt antibody, rabbit anti-phospho-Akt antibody, and rabbit anti-phospho-Akt substrate (RxRxxS*/T*) (23C8D2) antibody were from Cell Signaling Technology. Mouse anti-Arp3 antibody was from Santa Cruz Biotechnology. ACAP4 antibody was generated as described previously (Fang et al., 2006). Acapin antibody was generated by immunizing mouse with purified GST-tagged Acapin^1–179^ protein, and the specificity of Acapin antibody was tested by IB using HEK293T cell lysates. Mouse monoclonal anti-GFP antibody was purchased from BD Bioscience. Anti-Acapin-pS247 antibody was from YenZym. Phos-tag^TM^ Acrylamide was from Wako Pure Chemical Industries, Ltd. Alexa Fluor 488- and Alexa Fluor 647-conjugated phalloidin and Texas Red-X phalloidin were from Molecular Probes.

### siRNAs and plasmids

siRNA duplex targeting ACAP4 was obtained as described previously (Fang et al., 2006). siRNA duplexes targeting Acapin and Akt1, respectively, were purchased from Qiagen. HA-tagged Myr-Akt1 was kindly provided by Dr Jian Li at Harvard Medical School (Li et al., 2005). Human clone of Acapin gene was obtained from a testis cDNA library. FLAG-Acapin, FLAG-ACAP4, and the deletion mutants were constructed by inserting the corresponding gene sequences into the p3×FLAG-*myc*-CMV™-24 vector (Sigma). To construct site mutations, the cDNA was subcloned into the pEGFP vector (BD Biosciences). Bacterial recombinant proteins were constructed using pGEX-6P-1, pET-28a (Amersham Biosciences), and pETDuet-1 (Novagen) vectors. All constructs were validated by DNA sequencing.

### IP assay

The cells transfected with corresponding plasmids were collected and lysed with ice-cold buffer (50 mM Tris, pH 7.4, 100 mM NaCl, 1 mM EDTA, 1 mM DTT, and 0.2% Trition X-100 with protease inhibitors and phosphatase inhibitors). The whole-cell lysates (WCL) were resolved by SDS–PAGE and immunoblotted with indicated antibodies. For IP, the cell lysates were clarified by centrifugation at 13000 rpm for 20 min at 4°C and the supernatant was mixed with the indicated antibody for 4 h at 4°C followed by 2 h incubation with Protein A/G Sepharose Beads (Thermo Fisher Scientific). The beads were washed for three times with the lysis buffer (20 mM Tris, pH 7.4, 100 mM NaCl, 1 mM EDTA, 1 mM DTT, and 0.2% Trition X-100) and boiled in SDS–PAGE sample buffer. Subsequently, the immunoprecipitates were assayed by IB with indicated antibodies.

### Pull-down assay

Recombinant proteins including GST and His-tag fusion proteins were expressed in bacteria and purified as described previously (Hua et al., 2011). The pull-down assay was carried out by mixing purified proteins or cell lysates with GST fusion protein-bound glutathione beads followed by incubation in pull-down buffer (20 mM Tris–Cl, pH 7.4, 100 mM NaCl, 1 mM EGTA, 1 mM DTT, 0.25% Triton X-100, 1 mM PMSF, and protease inhibitor) for 3 h at 4°C. Then, the beads were washed with pull-down buffer for three times and boiled in SDS–PAGE sample buffer. The proteins were resolved by SDS–PAGE for CBB staining or IB analysis with indicated antibodies.

### ARF6 activity assay

Cells were transfected with Acapin for 24 h, starved in serum-free DMEM for 12 h, and then treated with 100 ng/ml EGF. Alternatively, cells were transfected with siRNAs for 72 h, deprived of serum for 12 h, and stimulated by EGF for 5 min. GGA pull-down assay to assess the relative level of active ARF6-GTP was performed as described previously (Santy and Casanova, 2001). The ARF6-GTP proteins bound to the beads were eluted in SDS–PAGE sample buffer and resolved by SDS–PAGE for IB analysis. Concurrently, the total ARF6 levels in WCL were quantified by IB analysis with the anti-ARF6 antibody.

### Wound healing assay and real-time imaging

For the wound healing assay, various types of cancer cells were cultured under optimal conditions until they reached 90% confluency. A linear scratch was then introduced into the confluent cell monolayer using a pipette tip. The cancer cells were allowed to migrate and close the wound over a period of 12 h, during which they were observed and captured using Olympus IX81 Microscope. The analysis of the images involved measuring the width of the scratch in each field of view at the initial time (0 h) and after 12 h of wound healing using ImageJ software. The migration percentage was calculated based on the reduction in scratch width over the 12-h period.

For the real-time cell migration assay, transfected cells were seeded on a glass-bottom culture dish (MatTek) coated with 10 μg/ml fibronectin. The cells were starved overnight and then stimulated with 100 ng/ml EGF. The cell movements were captured using a DeltaVision microscopy system (Applied Precision Inc.) with a 40× objective lens, at an interval of 1 frame every 6 min, and quantified using ImageJ software. The migration speed was calculated by dividing the total distance traveled by the total time elapsed, and the directional migration velocity was determined by dividing the straight-line distance from the starting point to the end point by the total time.

### Boyden chamber assay

HeLa cells were transfected with Acapin siRNAs and the scramble control. Forty-eight hours after transfection, cell suspension containing 1 × 10^5^ cells/ml in serum-free media was prepared and 100 μl cell suspension was added into the Corning-Costar 3422 transwell chamber with 500 μl DMEM (100 ng/ml EGF) in the lower wells. Then, the invasion assay was performed at 37°C for 16 h according to the manufacturer's instructions. Nonmigratory cells were removed from the chamber using cotton-tipped swabs. Subsequently, the migrated cells through the chamber were fixed, stained with crystal violet, and imaged under a DeltaVision microscopy system.

### Yeast two-hybrid assay

Yeast two-hybrid assays were performed as previously described (Liu et al., 2007). Briefly, the plasmids of BD-tagged protein and AD-tagged protein were transfected into yeast strain AH109. Then, the yeast cells were grown on SD medium with X-α-Gal but lacking Leu, Trp, His, and Ade.

### Immunofluorescence

The cells were transfected with the indicated plasmids. Twenty-four hours after transfection, the cells were serum-deprived for 6 h followed by stimulation with 100 ng/ml EGF for 5 min in the presence or absence of MK2206 or TCN. The cells were fixed with 5 min in 3.7% paraformaldehyde and permeabilized for 2 min with 0.1% Triton X-100 in PBS (PBST). Coverslips were blocked with PBST with 1% bovine serum albumin for 1 h, stained with primary antibodies for 1 h, washed with PBST for three times, and then incubated with cross-absorbed secondary antibodies. Coverslips were visualized under a DeltaVision deconvolution microscope.

### Statistics

All data were obtained from at least three independent experiments. Graphs were made using GraphPad Prism 8 and statistical tests were performed using Student's *t*-test (unpaired two-tailed) for comparisons between two groups and one-way analysis of variance (ANOVA) for multiple comparisons. All statistical parameters are described in the figure legend of each figure, including sample size, error calculations, and *P*-values. *P* < 0.05 was considered to be significant. No statistical method was used to predetermine the sample size. No data were excluded from the analyses. Results are shown as mean ± SEM.

## Supplementary Material

mjaf010_Supplemental_File

## Notes

**Author contributions:** X.Y. conceived the project. C.W., J.Z., T.L., S.D., Y.C., and M.M. performed the experiments and analyzed the data. C.W., F.Y., and X.Y. wrote the manuscript. T.U., D.W., Y.C., X.C., S.-Y.C., X.D., and X.Y. reviewed the manuscript. All authors have commented and approved the manuscript.

## References

[bib1] Chen Y., Wang D., Guo Z. et al. (2011). Rho kinase phosphorylation promotes ezrin-mediated metastasis in hepatocellular carcinoma. Cancer Res. 71, 1721–1729. 21363921 10.1158/0008-5472.CAN-09-4683 PMC3119000

[bib2] Ding X., Deng H., Wang D. et al. (2010). Phospho-regulated ACAP4–Ezrin interaction is essential for histamine-stimulated parietal cell secretion. J. Biol. Chem. 285, 18769–18780. 20360010 10.1074/jbc.M110.129007 PMC2881800

[bib3] Donaldson J.G., Jackson C.L. (2011). ARF family G proteins and their regulators: roles in membrane transport, development and disease. Nat. Rev. Mol. Cell Biol. 12, 362–375. 21587297 10.1038/nrm3117 PMC3245550

[bib4] Du S., Song X., Li Y. et al. (2020). Celastrol inhibits ezrin-mediated migration of hepatocellular carcinoma cells. Sci. Rep. 10, 11273. 32647287 10.1038/s41598-020-68238-1 PMC7347585

[bib5] Fang Z., Miao Y., Ding X. et al. (2006). Proteomic identification and functional characterization of a novel ARF6 GTPase-activating protein, ACAP4. Mol. Cell. Proteomics 5, 1437–1449. 16737952 10.1074/mcp.M600050-MCP200

[bib6] Feng H., Lopez G.Y., Kim C.K. et al. (2014). EGFR phosphorylation of DCBLD2 recruits TRAF6 and stimulates AKT-promoted tumorigenesis. J. Clin. Invest. 124, 3741–3756. 25061874 10.1172/JCI73093 PMC4151226

[bib7] Gillingham A.K., Munro S. (2007). The small G proteins of the Arf family and their regulators. Annu. Rev. Cell Dev. Biol. 23, 579–611. 17506703 10.1146/annurev.cellbio.23.090506.123209

[bib8] Ha V.L., Bharti S., Inoue H. et al. (2008). ASAP3 is a focal adhesion-associated Arf GAP that functions in cell migration and invasion. J. Biol. Chem. 283, 14915–14926. 18400762 10.1074/jbc.M709717200 PMC2397480

[bib9] Hemmings B.A., Restuccia D.F. (2012). PI3K–PKB/Akt pathway. Cold Spring Harb. Perspect. Biol. 4, a011189. 22952397 10.1101/cshperspect.a011189 PMC3428770

[bib10] Hu Z., Du J., Yang L. et al. (2012). GEP100/Arf6 is required for epidermal growth factor-induced ERK/Rac1 signaling and cell migration in human hepatoma HepG2 cells. PLoS One 7, e38777. 22701712 10.1371/journal.pone.0038777 PMC3372492

[bib11] Hua S., Wang Z., Jiang K. et al. (2011). CENP-U cooperates with Hec1 to orchestrate kinetochore–microtubule attachment. J. Biol. Chem. 286, 1627–1638. 21056971 10.1074/jbc.M110.174946 PMC3020771

[bib12] Huang Y., Lin L., Liu X. et al. (2019). BubR1 phosphorylates CENP-E as a switch enabling the transition from lateral association to end-on capture of spindle microtubules. Cell Res. 29, 562–578. 31201382 10.1038/s41422-019-0178-z PMC6796941

[bib13] Ismail S.A., Vetter I.R., Sot B. et al. (2010). The structure of an Arf–ArfGAP complex reveals a Ca^2+^ regulatory mechanism. Cell 141, 812–821. 20510928 10.1016/j.cell.2010.03.051

[bib14] Jackson T.R., Brown F.D., Nie Z. et al. (2000). ACAPs are arf6 GTPase-activating proteins that function in the cell periphery. J. Cell Biol. 151, 627–638. 11062263 10.1083/jcb.151.3.627 PMC2185579

[bib15] Jiang H., Wang W., Zhang Y. et al. (2015). Cell polarity kinase MST4 cooperates with cAMP-dependent kinase to orchestrate histamine-stimulated acid secretion in gastric parietal cells. J. Biol. Chem. 290, 28272–28285. 26405038 10.1074/jbc.M115.668855 PMC4653683

[bib16a] Krugmann S., Anderson K.E., Ridley S.H. et al. (2002). Identification of ARAP3, a novel PI3K effector regulating both Arf and Rho GTPases, by selective capture on phosphoinositide affinity matrices. Mol. Cell 9, 95–108. 11804589 10.1016/s1097-2765(02)00434-3

[bib16aaa] Leemans C.R., Braakhuis B.J., Brakenhoff R.H et al. (2011). The molecular biology of head and neck cancer. Nat. Rev. Cancer 11, 9–22. 21160525 10.1038/nrc2982

[bib16] Li J., Ballif B.A., Powelka A.M. et al. (2005). Phosphorylation of ACAP1 by Akt regulates the stimulation-dependent recycling of integrin β1 to control cell migration. Dev. Cell 9, 663–673. 16256741 10.1016/j.devcel.2005.09.012

[bib17] Liu D., Ding X., Du J. et al. (2007). Human NUF2 interacts with centromere-associated protein E and is essential for a stable spindle microtubule–kinetochore attachment. J. Biol. Chem. 282, 21415–21424. 17535814 10.1074/jbc.M609026200

[bib18] Liu P., Gan W., Guo C. et al. (2015). Akt-mediated phosphorylation of XLF impairs non-homologous end-joining DNA repair. Mol. Cell 57, 648–661. 25661488 10.1016/j.molcel.2015.01.005 PMC4336609

[bib19] Liu X., Liu X., Wang H. et al. (2020). Phase separation drives decision making in cell division. J. Biol. Chem. 295, 13419–13431. 32699013 10.1074/jbc.REV120.011746 PMC7521646

[bib20] Ma Z., Nie Z., Luo R. et al. (2007). Regulation of Arf6 and ACAP1 signaling by the PTB-domain-containing adaptor protein GULP. Curr. Biol. 17, 722–727. 17398097 10.1016/j.cub.2007.03.014 PMC1930157

[bib21] Manning B.D., Cantley L.C. (2007). AKT/PKB signaling: navigating downstream. Cell 129, 1261–1274. 17604717 10.1016/j.cell.2007.06.009 PMC2756685

[bib22] Matsukawa J., Nakayama K., Nagao T. et al. (2003). Role of ADP-ribosylation factor 6 (ARF6) in gastric acid secretion. J. Biol. Chem. 278, 36470–36475. 12860984 10.1074/jbc.M305444200

[bib23] Mizuno-Yamasaki E., Rivera-Molina F., Novick P. (2012). GTPase networks in membrane traffic. Annu. Rev. Biochem. 81, 637–659. 22463690 10.1146/annurev-biochem-052810-093700 PMC3708692

[bib24] Morishige M., Hashimoto S., Ogawa E. et al. (2008). GEP100 links epidermal growth factor receptor signalling to Arf6 activation to induce breast cancer invasion. Nat. Cell Biol. 10, 85–92. 18084281 10.1038/ncb1672

[bib25] Mullen M., Yang F., Cao J. et al. (2021) Syntelin inhibits triple-negative breast cancer cell proliferation and metastasis. J. Mol. Cell Biol. 13, 834–837. 10.1093/jmcb/mjab054 PMC8782585 34450654

[bib26] Okabe H., Furukawa Y., Kato T. et al. (2004). Isolation of development and differentiation enhancing factor-like 1 (DDEFL1) as a drug target for hepatocellular carcinomas. Int. J. Oncol. 24, 43–48. 14654939

[bib27] Pasqualato S., Menetrey J., Franco M. et al. (2001). The structural GDP/GTP cycle of human Arf6. EMBO Rep. 2, 234–238. 11266366 10.1093/embo-reports/kve043 PMC1083839

[bib28] Qiao M., Sheng S., Pardee A.B. (2008). Metastasis and AKT activation. Cell Cycle 7, 2991–2996. 18818526 10.4161/cc.7.19.6784

[bib29] Radhakrishna H., Klausner R.D., Donaldson J.G. (1996). Aluminum fluoride stimulates surface protrusions in cells overexpressing the ARF6 GTPase. J. Cell Biol. 134, 935–947. 8769418 10.1083/jcb.134.4.935 PMC2120964

[bib30] Randazzo P.A., Inoue H., Bharti S. (2007). Arf GAPs as regulators of the actin cytoskeleton. Biol. Cell. 99, 583–600. 17868031 10.1042/bc20070034

[bib31] Santy L.C., Casanova J.E. (2001). Activation of ARF6 by ARNO stimulates epithelial cell migration through downstream activation of both Rac1 and phospholipase D. J. Cell Biol. 154, 599–610. 11481345 10.1083/jcb.200104019 PMC2196419

[bib32] Scheid M.P., Woodgett J.R. (2001). PKB/AKT: functional insights from genetic models. Nat. Rev. Mol. Cell Biol. 2, 760–768. 11584303 10.1038/35096067

[bib33] Schweitzer J.K., Sedgwick A.E., D'Souza-Schorey C. (2011). ARF6-mediated endocytic recycling impacts cell movement, cell division and lipid homeostasis. Semin. Cell Dev. Biol. 22, 39–47. 20837153 10.1016/j.semcdb.2010.09.002 PMC3457924

[bib34] Song X., Liu W., Yuan X. et al. (2018). Acetylation of ACAP4 regulates CCL18-elicited breast cancer cell migration and invasion. J. Mol. Cell Biol. 10, 559–572 30395269 10.1093/jmcb/mjy058 PMC6692856

[bib35] Song X., Wang W., Wang H. et al. (2020). Acetylation of ezrin regulates membrane–cytoskeleton interaction underlying CCL18-elicited cell migration. J. Mol. Cell Biol. 12, 424–437. 31638145 10.1093/jmcb/mjz099 PMC7333480

[bib36] Song X., Yang F., Liu X. et al. (2021). Dynamic crotonylation of EB1 by TIP60 ensures accurate spindle positioning in mitosis. Nat. Chem. Biol. 17, 1314–1323. 34608293 10.1038/s41589-021-00875-7

[bib37] Spang A., Shiba Y., Randazzo P.A. (2010). Arf GAPs: gatekeepers of vesicle generation. FEBS Lett. 584, 2646–2651. 20394747 10.1016/j.febslet.2010.04.005 PMC2878913

[bib38] Wei W., Zheng B., Zheng S. et al. (2023) The Cdc42 GAP Rga6 promotes monopolar outgrowth of spore. J. Cell Biol. 222, e202202064. 36355349 10.1083/jcb.202202064 PMC9652770

[bib39] Xue G., Hemmings B.A. (2013). PKB/Akt-dependent regulation of cell motility. J. Natl Cancer Inst. 105, 393–404. 23355761 10.1093/jnci/djs648

[bib40] Xue Y., Zhou F., Zhu M. et al. (2005). GPS: a comprehensive www server for phosphorylation sites prediction. Nucleic Acids Res. 33, W184–W187. 15980451 10.1093/nar/gki393 PMC1160154

[bib41] Yu X., Wang F., Liu H. et al. (2011). ACAP4 protein cooperates with Grb2 protein to orchestrate epidermal growth factor-stimulated integrin beta1 recycling in cell migration. J. Biol. Chem. 286, 43735–43747. 22027826 10.1074/jbc.M111.278770 PMC3243555

[bib42] Yuan X., Yao P.Y., Jiang J. et al. (2017). MST4 kinase phosphorylates ACAP4 protein to orchestrate apical membrane remodeling during gastric acid secretion. J. Biol. Chem. 292, 16174–16187. 28808054 10.1074/jbc.M117.808212 PMC5625048

[bib43] Zhang H., Zha X., Tan Y. et al. (2002). Phosphoprotein analysis using antibodies broadly reactive against phosphorylated motifs. J. Biol. Chem. 277, 39379–39387. 12151408 10.1074/jbc.M206399200

[bib44] Zhao X., Wang D., Liu X. et al. (2013). Phosphorylation of the bin, amphiphysin, and RSV161/167 (BAR) domain of ACAP4 regulates membrane tubulation. Proc. Natl Acad. Sci. USA 110, 11023–11028. 23776207 10.1073/pnas.1217727110 PMC3703971

