# Supplementary material for: Akt-elicited phosphorylation of Acapin steers cell migration
Source: J Mol Cell Biol. 2025 Mar 13;17(3):mjaf010. doi: 10.1093/jmcb/mjaf010 (PMC12570880; doi:10.1093/jmcb/mjaf010)
Supplement: mjaf010_Supplemental_File [file mjaf010_supplemental_file.pdf]

Supplementary Figure S1

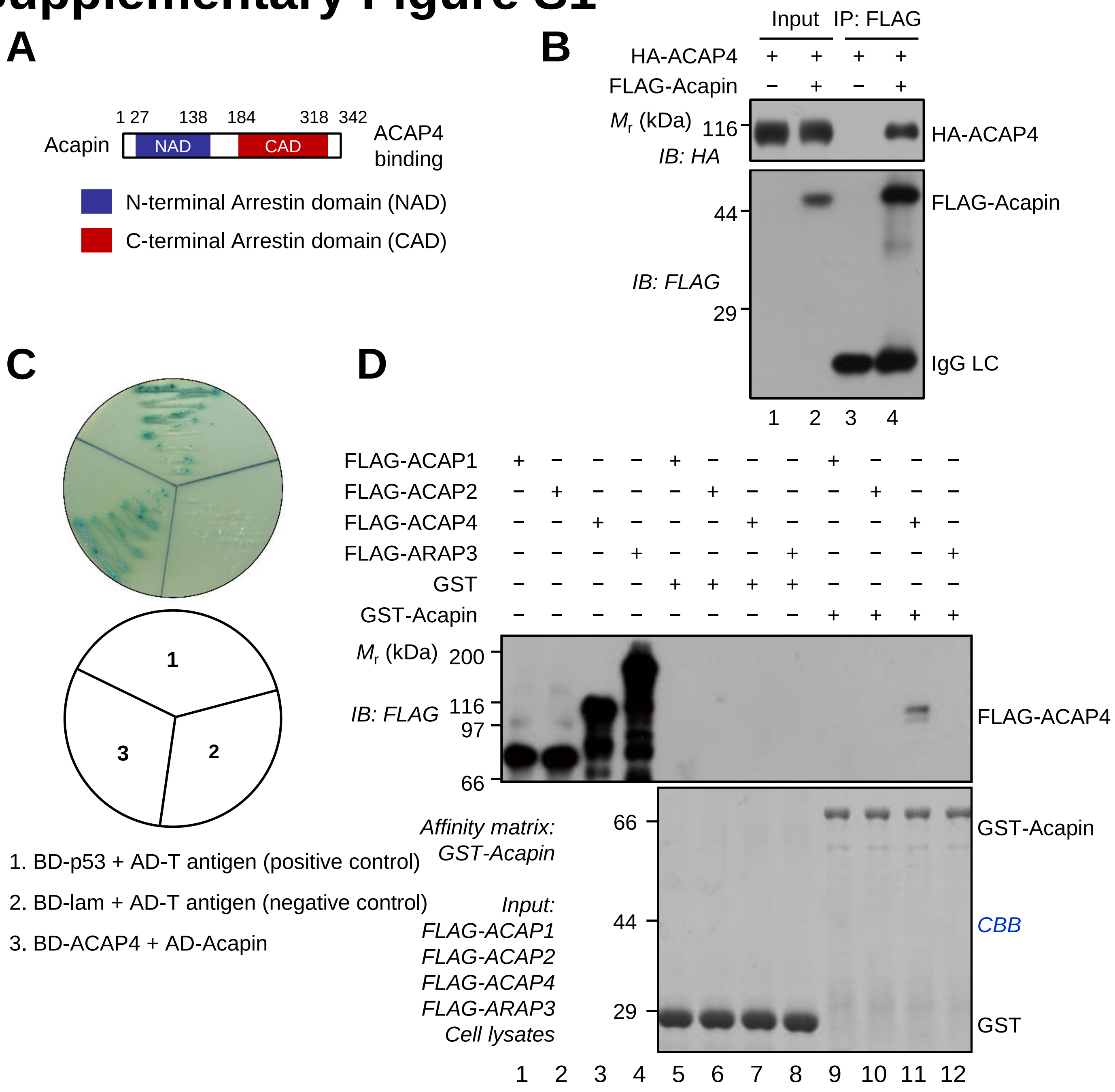

Supplementary Figure S1. Schematic drawing of Acapin domain structure and characterization.

A. Acapin contains two putative arrestin-like domains.

B. Acapin associates with ACAP4 in cells. HEK293T cells transfected with FLAG-Acapin and HA-ACAP4 were lysed and incubated with anti-FLAG M2 affinity matrix. Immunoprecipitates were resolved by SDS-PAGE and detected by immunoblotting (IB) with anti-HA antibody (upper) and anti-FLAG antibody (lower). Lane 1 and 2, the cell lysates before incubating with anti-FLAG M2 beads, lane 3 and 4, the anti-FLAG immunoprecipitates.

C. Yeast two-hybrid assays for the interaction between ACAP4 and Acapin *in vivo*.

D. GST pull-down assay for the interaction of Acapin with ACAP1, ACAP2, ACAP4 and ARAP3. Recombinant GST-Acapin proteins purified on glutathione beads was used as an affinity matrix for absorbing FLAG-tagged ARFGAPs expressed in HEK293T cells.

Supplementary Figure S2

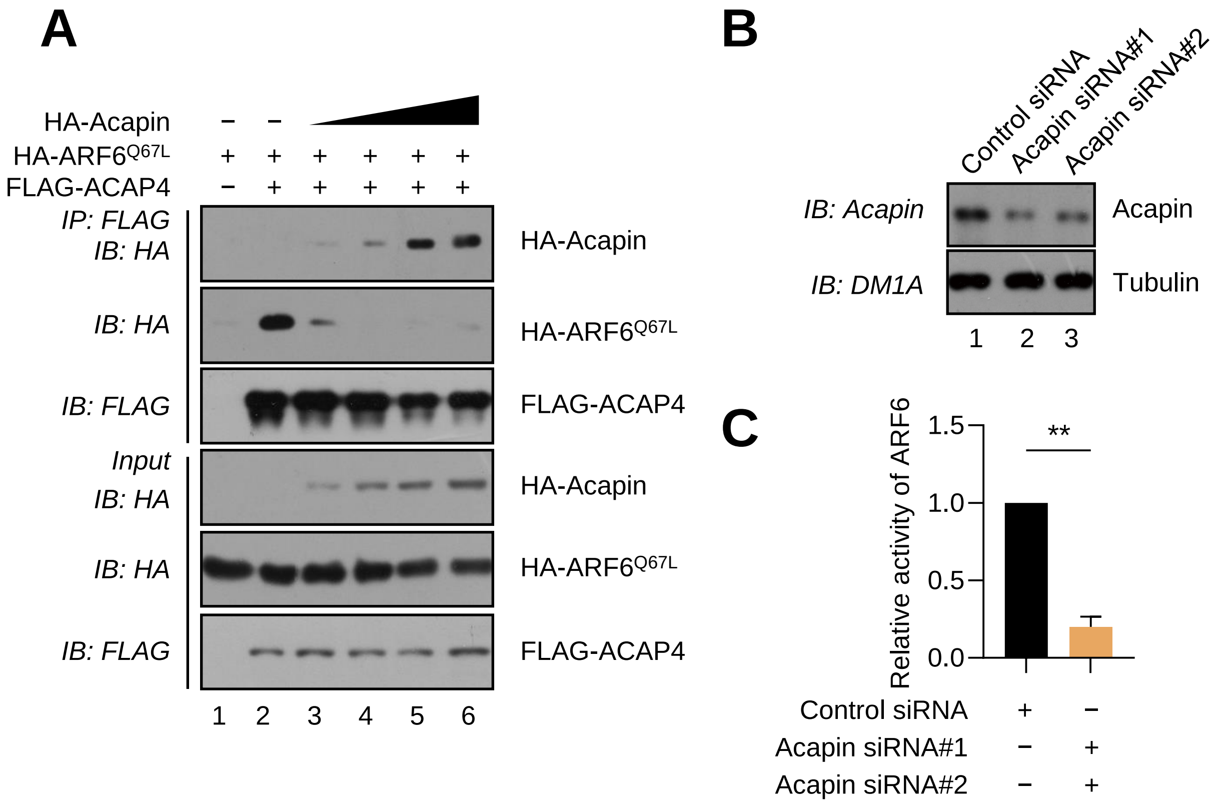

Supplementary Figure S2. Characterization of Acapin-ACAP4-ARF6 interactions

A. Overexpression of Acapin disrupts the ACAP4-ARF6 interaction. HEK293T cells were transfected with constitutively active ARF6^{Q67L}, HA-Acapin, and FLAG-ACAP4, IP-IB assay was used to determine the effect of Acapin on binding ability of FLAG-ACAP4 to HA-ARF6^{Q67L}.

B. Western blotting analysis of the knockdown efficiency of Acapin siRNAs.

C. Statistical analysis of relative ARF6 activity in Acapin-depleted cells. Data are presented as mean ± SEM, n = 3, $**p < 0.01$.

Supplementary Figure S3

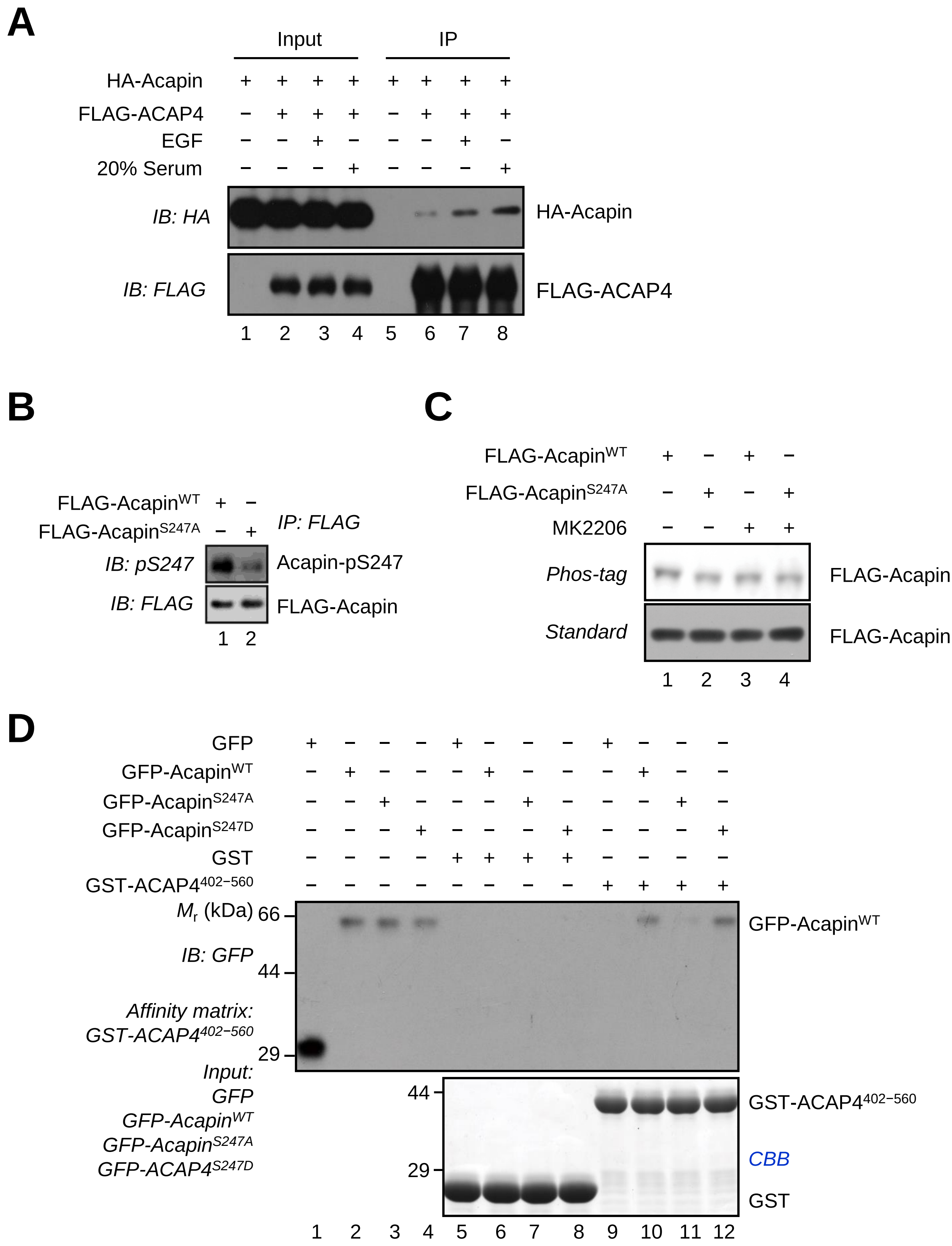

Supplementary Figure S3. Phosphorylation at Ser247 of Acapin mediates its association with the GAP domain of ACAP4.

A. HeLa cells were transiently co-transfected to express GFP-Acapin and FLAG-ACAP4. The cells were then deprived of serum for 6 h followed by EGF or serum stimulation for 5 min. FLAG immunoprecipitation assay was conducted to determine the binding of Acapin and ACAP4.

B. HeLa cells transfected with FLAG-Acapin wild type and S247A mutant were subjected to FLAG immunoprecipitation. The immunoprecipitates were analyzed by IB with Acapin-pS247 antibody.

C. Whole cell lysates of HeLa cells transiently transfected to express FLAG-Acapin wild type and S247A mutant were resolved on an SDS-PAGE gel containing phos-tag acrylamide (phos-tag). A standard SDS-PAGE gel was used as control.

D. Recombinant GST-ACAP4$^{402-560}$ was purified and immobilized on glutathione beads. The beads were incubated with GFP-Acapin wild type and mutants from HEK293T cells. The bound proteins was subjected to IB analysis with an anti-GFP antibody.

Supplementary Figure S4

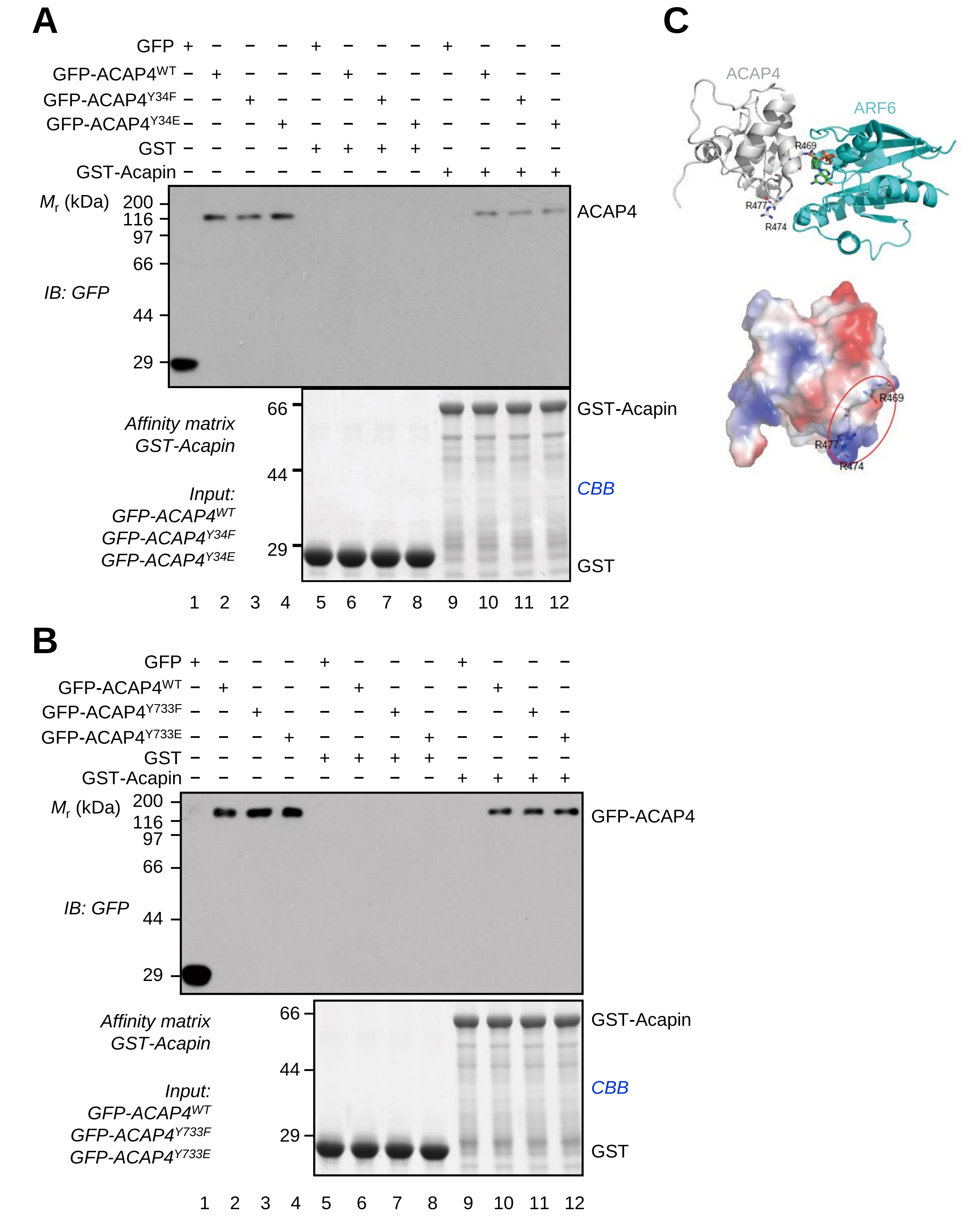

Supplementary Figure S4. Phosphorylation of ACAP4 at Y34 and Y733 does not affect its association with Acapin. Phosphorylation at Y34 of ACAP4 does not affect its interaction with Acapin. GST-Acapin on glutathione beads were incubated with GFP-ACAP4 wild type and mutants from HEK293T cells. The proteins bound to agarose beads were analyzed by CBB and IB using anti-GFP antibody.

B. Phosphorylation at Y733 of ACAP4 does not affect its interaction with Acapin. GST-Acapin on glutathione beads were incubated with GFP-ACAP4 wild type and mutants from HEK293T cells. The proteins bound to agarose beads were analyzed by CBB and IB using anti-GFP antibody.

C. Upper: Ribbon representation of the ARF6-ACAP4 complex (PDB code: 3LVR). The ACAP4-GAP domain is colored in grey, and ARF6 is colored in cyan. Positively charged residues located in ARF6-ACAP4 interface are labeled as sticks. Lower: An electrostatic potential surface presentation of ACAP4, in which positively charged, negatively charged and neutral areas are presented in blue, red and white, respectively. The positively charged groove nearing the binding interface of ARF6-ACAP4 is highlighted in a red circle. The structures were prepared with PyMOL.

Supplementary Figure S5

A

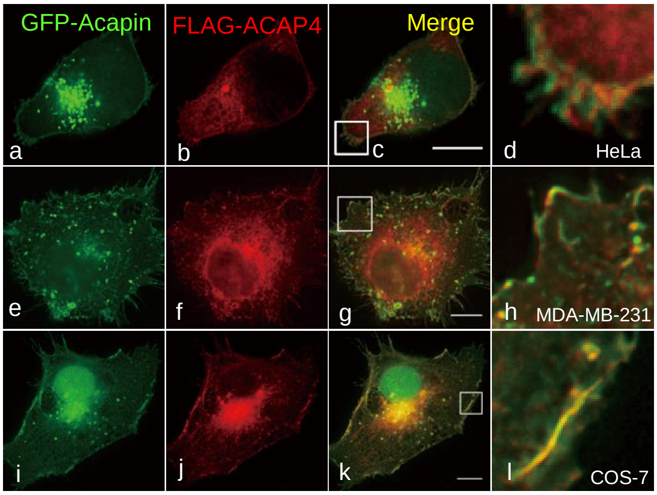

B

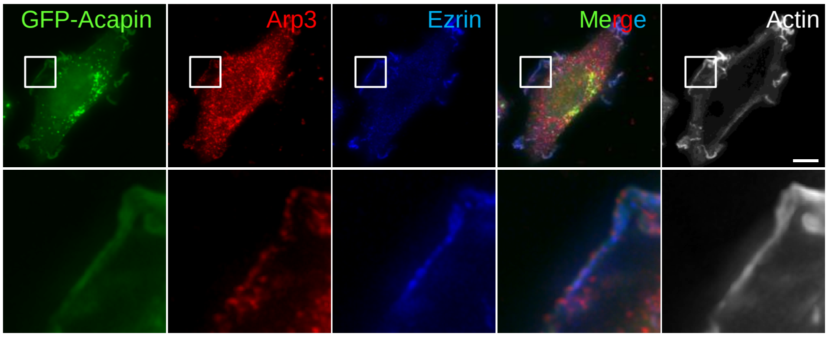

Supplementary Figure S5. Acapin colocalizes with ACAP4 at plasma membrane.
A. HeLa, MDA-MB-231 and COS-7 cells were cotransfected with GFP-Acapin and FLAG-ACAP4 for 24 h. Cells were then fixed, permeabilized for visualization of GFP-Acapin (green) and stained with FLAG antibody (FLAG-ACAP4, red). Scale bars, 10 μm. Panels d, h and l are magnified portion of merge panels c, g and k indicated by white boxes.
B. HeLa cells transfected with GFP-Acapin were fixed, permeabilized for visualization of GFP-Acapin (green) and stained with Arp3 antibody (red), Ezrin antibody (blue), Alexa Fluor 647 Phalloidin (white). Scale bar, 10 μm. Bottom panels are magnified portion of top panels indicated by white boxes.

Supplementary Figure S6

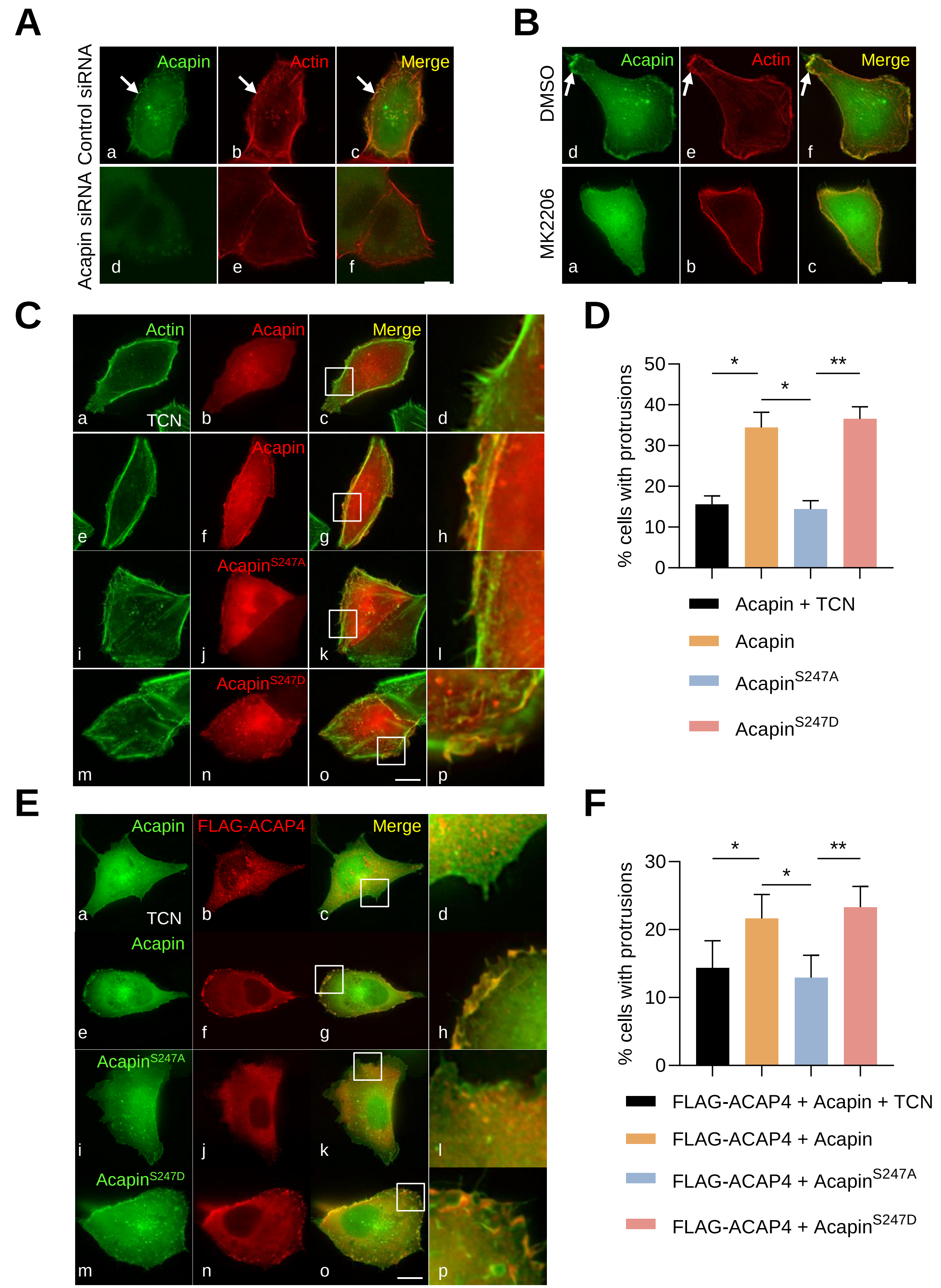

Supplementary Figure S6. Colocalization of Acapin and ACAP4 at plasma membrane is dependent on Akt phosphorylation.

A. Depletion of Acapin suppressed cell protrusions upon EGF stimulation. HeLa cells transfected with Acapin siRNAs were deprived of serum for 6 h followed by EGF stimulation for 5 min. Cells were then fixed and stained using phalloidin to detect actin (red). Scale bars, 10 μm.

B. HeLa cells were deprived of serum for 6 h and stimulated with EGF (100 ng/ml) for 5 min. TCN was added into cells 2 h before EGF stimulation. Cells were then fixed and stained with an anti-Acapin antibody (green) and phalloidin to detect actin (red). Scale bars, 10 μm.

C. HeLa cells were transfected with GFP-Acapin wild type and mutants for 24 h. The cells were then deprived of serum for 6 h and stimulated with EGF (100 ng/ml) for 5 min. TCN was added into cells 2 h before EGF stimulation. Cells were then fixed and stained with phalloidin to detect actin (green). Scale bars, 10 μm.

D. Quantitative analyses of protrusions of the cells as shown in (C). Data are mean ± SEM. $*p < 0.05$; $**p < 0.01$.

E. HeLa cells were co-transfected with FLAG-ACAP4 and GFP-Acapin wild type and mutants for 24 h. The cells were then deprived of serum for 6 h and stimulated with EGF (100 ng/ml) for 5 min. TCN was added into cells 2 h before EGF stimulation. Cells were then fixed for imaging. Scale bars, 10 μm.

F. Quantitative analyses of protrusions of the cells as shown in (E). Data are mean ± SEM. $*p < 0.05$; $**p < 0.01$.

Supplementary Figure S7

A

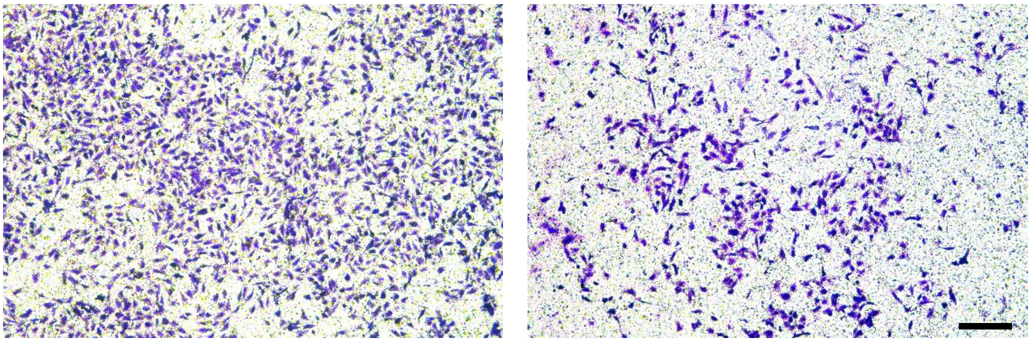

Supplementary Figure S7. Acapin orchestrates EGF-elicited HeLa cell invasion

A. HeLa cells were transfected with control siRNA or Acapin siRNA for 48 h. Cells were then seeded to the Boyden chamber assay. Invasive cells through the membrane were stained with crystal violet, and the images were taken by light microscope. Scale bar, 200 μm.
